# Is Grasping Impaired in Hemispatial Neglect?

**DOI:** 10.1155/2002/495854

**Published:** 2002-08-01

**Authors:** Monika Harvey, Stephen R. Jackson, Roger Newport, Tanja Krämer, D. Llewlyn Morris, Lindsay Dow

**Affiliations:** ^1^ Department of Psychology University of Glasgow Glasgow UK; ^2^ School of Psychology University of Nottingham Nottingham NG7 2RD UK; ^3^ Centre for Perception Attention and Motor Sciences University of Wales Bangor Gwynedd LL57 2DG UK; ^4^ Care of the Elderly Frenchay Hospital University of Bristol BS8 1PN UK

## Abstract

Patients with right unilateral cerebral stroke, four of which showed acute hemispatial neglect, and healthy aged-matched controls were tested for their ability to grasp objects located in either right or left space at near or far distances. Reaches were performed either in free vision or without visual feedback from the hand or target object. It was found that the patient group showed normal grasp kinematics with respect to maximum grip aperture, grip orientation, and the time taken to reach the maximum grip aperture. Analysis of hand path curvature showed that control subjects produced straighter right hand reaches when vision was available compared to when it was not. The right hemisphere lesioned patients, however, showed similar levels of curvature in each of these conditions. No behavioural differences, though, could be found between right hemisphere lesioned patients with or without hemispatial neglect on either grasp parameters, path deviation or temporal kinematics.

## 1. Introduction

Prehension requires the integration of visual and somatosensory information into a co-ordinated motor plan for transporting the arm to a target while shaping the hand to match the target geometry. The role of vision in grasping is not only to activate proper schemas and specify the composition of the fingers but also to determine the relative positions of the hand and the object to be grasped, as accurate positioning of the fingers on the object surface are a prerequisite for the subsequent handling and manipulation of the object. The formation of the grasp before contact with the object is therefore the critical factor that governs the movements of the other segments of the upper limb during the reach (see [20] for review). Although reach and grasp can be described as separate subsystems [18,19], studies of reaching in isolation from grasping ignore many of these key aspects of its control.

The areas of the human brain that control prehension are not defined in detail and patients with focal lesions primarily affecting prehension seem to be rare [18,19]. One of the first researchers to highlight a specific grasping impairment as a result of bilateral posterior parietal lesions was Rudolf Bálint [1]. He described the disorder as 'optic ataxia', a specific deficit of the visual control of movement unrelated to motor, somatosensory, visual acuity or visual field deficits. More recent studies into the visuomotor behaviour of humans have confirmed Balint's observation. Jeannerod [17] and Perenin and Vighetto [31] reported patients with parietal lesions whose reaching movements proved inaccurate, often erred in one direction and were also kinematically altered, with increased movement durations and lower peak velocities. Additionally, during prehension of objects the finger grip proved too wide, with either no or poor preshaping and the grasp closing on contact with the object only, a finding replicated more recently by Jakobson et al. [16]. It thus seems that lesions that do affect goal-directed behaviour are most commonly centred in the posterior parietal lobe and indeed imaging studies [11,37] confirm this. In fact, a recent study by Binkofski et al. [2] that investigated grasping behaviour in patients with parietal le-

ISSN 0953-4180/01/02/$8.00 © 2001/2002 – IOS Press. All rights reserved sions showed that patients with lesions of the anterior part of the intraparietal sulcus had distinct impairments in grasping. Moreover, these lesion data were supplemented by functional MRI data showing specific activation of this area during grasping in normal subjects.

Patients suffering from hemispatial neglect after right hemisphere lesions, usually to the parietal lobe (although frontal and subcortical structures have been shown to be involved as well [38]), unsurprisingly also show a characteristic disturbance of visuospatial behaviour. They typically demonstrate a deficient response to stimuli located contralaterally to the lesion and fail to explore the contralesional space with either eye or limb movements. Despite these striking visuospatial impairments the substantial majority of existing research into hemispatial neglect has been concerned with the biased selection of ipsi-over contralesional input (see [33] for review). Although a number of investigations have looked at reaction and movement times towards points and objects in the contralesional hemispace, and have generally found these to be increased in hemispatial neglect (see [25] for review), only very few studies to date have examined the visuomotor output patterns of these patients, i.e., their reaching and grasping parameters in terms of reach trajectory and grasp kinematics. This lack of investigations is striking since there is now abundant evidence that perceptual processes in humans and many other species are tightly constrained by the kinds of responses they control (see [26,27] for review). Studies that have in fact directly investigated pointing and grasping behaviour in hemispatial neglect have revealed partly diverging results:

In 1990, Goodale and colleagues [9] investigated visually guided pointing in right hemisphere lesioned patients who had recovered from neglect. Patients were asked to point either midway between two lights or directly on top of a single light. It was found that all patients made large rightward directional errors at the outset of the reach. These initial errors were observed not only in bisection, but also in simple pointing; however they were more poorly corrected in the bisection task, so that the final rightward errors remained much larger than those seen in pointing. A related study on pointing and bisecting (this time including open loop reaches) was run by Harvey et al. [12] using a wider sample of right-and-left hemisphere lesioned patients, none of them revealing signs of hemispatial neglect at the time of testing (only two right hemisphere lesioned patients had ever shown any sign of neglect). In contrast to the results of Goodale et al. rightward biases in the reach trajectory, as well as a larger terminal error for the right hemisphere lesioned patients, were found only in the absence of visual feedback but not in the closed loop pointing and bisection tasks. Although one might be tempted to explain the differences between the two studies in terms of patient population (Goodale's patients had recovered from neglect but Harvey's patients had never shown any sign of it) this interpretation seems unlikely in view of a recent result by Karnath and colleagues. For the first time Karnath et al. [22] actually tested acute neglect patients, as well as right hemisphere lesioned patients without neglect, with a simple pointing task and found no evidence of a rightward bias in the reach trajectory neither in the closed nor the open loop condition. In fact, neither patient group varied from the healthy controls in terms of either reach deviation or final accuracy. This finding seems surprising in the light of the severe visuospatial disturbance these patients generally experience. However, a similarly good result was found by Chieffi and colleagues [4] in a grasping experiment in which a recovered neglect patient showed normal reaching and grasping towards single objects. Only when distractor objects where presented simultaneously to the right of the target, did she show a rightward deviation of the wrist trajectory, her grip aperture, however, was never affected. This last finding was also repeated in an experiment by Pritchard et al. [32] whose acute neglect patient showed normal grip aperture towards targets of different sizes. Unfortunately reach trajectory was not reported in this study.

The main objective of the present study was thus to clarify the issue of goal-directed behaviour in hemispatial neglect. Are these patients impaired while grasping for single objects in right and left space? It is inherently difficult to accurately assess hemispatial reaching and grasping differences in a neglect population as hemiplegia prevents left hand movements in almost all the cases. However, it has been clearly shown that ipsi-and contralesional reaches differ in terms of their mechanics [3]. It is thus difficult to disentangle these mechanical hemispatial from visuomotor effects unless reaches are performed to identical positions under different feedback conditions [12,22,15]. Both the Chieffi et al. [4] and Pritchard et al. [32] studies indicate that there might not be hemispatial grasping effects but then only single cases were studied and none of the conditions required an open loop grasp. Secondly, even if the grasp component is intact, will the path curvature prove unbiased as shown by Karnath and colleagues? We thus asked four neglect patients to grasp towards objects in right and left hemispace, either with or without visual feedback of the hand and measured their grasp kinematics as well as their path curvature and velocity profiles. Performance was compared to right hemisphere lesioned patients without neglect as well as to matched healthy controls.

## 2. Method

### 2.1. Subjects

Two groups of subjects were tested: 7 patients with unilateral right hemisphere infarct [R CVA] (mean age = 68, SD = 9.7, 5 male, 2 female), four of which showed evidence of hemispatial neglect at the time of testing when assessed with the BIT [40] and 5 normal control subjects (mean age = 68, SD. = 5.7). Ethical approval had been given by Frenchay Healthcare Trust and all subjects gave their informed consent to participate in the experiment prior to testing. All subjects in the two patient groups had suffered cerebrovascular accidents within the previous 43 months of testing. CT scans were available on all the patients, and none of these aroused any suspicion of bilateral damage. All subjects were right handed and it was ensured that none of the subjects in the control group had any appreciable medical, neurological or psychiatric history. There were no significant differences between the control group and the patient group in age or education (mean R CVA = 9.8 years, SD = 5.8; mean controls = 10.6 years, SD = 1.4). Clinical and neuropsychological details of all the patients are listed in Table 1.

### 2.2. Apparatus and procedures

Subjects were seated at a 1000 mm square table and – from a fixed starting position, that was indicated by a T-shaped mark 3 cm tall by 4 cm wide, placed centrally in front of them – executed prehension movements toward target objects presented in the frontal plane (red wooden dowels 50 mm high with a diameter of 22.5 mm). None of the patients could use their contralesional hand so only ipsilesional reaches were tested. The control subjects used their right hand also.

Targets were placed at one of four locations, two to the left and two to the right equidistant (100 mm) from the subject's mid-saggittal axis positioned at either 200 mm (near) or 250 mm (far) from the starting position. Target size was not manipulated as this had been done in previous studies with no significant effects [4,32] and would have resulted in too many trials and independent variables.

Subjects began each trial with their hand placed flat upon the starting position, oriented along the saggital plane, and with their thumb closed against their index finger. All subjects executed four types of prehension movements: unimanual near and far reaches in right hemispace and unimanual near and far reaches in left hemispace.

To control viewing conditions, subjects wore a set of glasses that were fitted with liquid crystal lenses throughout the experiment (Plato Systems, Translucent Technologies Inc.). It is important to note that occlusion with these lenses does not significantly decrease levels of illumination to the eye. Each trial commenced with the glasses clearing. On half the trials the glasses remained clear throughout the reach (binocular viewing). On the remaining trials vision was occluded again at movement onset, i.e., as soon as the subject lifted the fingers from the start block (open loop condition).

Each subject completed six trials towards each of the four target locations (24 trials) in both the open and closed loop conditions thus performing a total of 48 trials. Testing was done in four blocks of 12 trials with blocked presentation of open and closed loop trials to avoid fatigue and allow breaks between blocks. Order of presentation of trials for each block was individually randomised for each subject and presentation of blocks balanced across patients and controls. Subjects were instructed to reach at normal speed and maintain accuracy. A brief practice session for both open loop and binocular viewing conditions was conducted prior to the presentation of the experimental sessions.

### 2.3. Movement recording and data analysis

Hand movements were recorded using a MacReflex infra-red motion-analysis system (Qualisys Inc.) with a sampling rate of 50 Hz. 5 mm $\times$ 5 mm reflective markers were placed on the distal portion of the thumbnail, on the distal portion of the index finger and on the wrist of the right hand. An additional marker was also fixed to the target object. The 3D spatial co-ordinates of these markers were analysed off-line using custom software with Labview (National Instruments Inc.) and Matlab (The Mathworks Inc.) programming environments. Data were low pass filtered using an 4th-order Butterworth filter (cut-off frequency of 10 Hz).

Table 1
Clinical data for the three patient groups. BIT-Score:Total score achieved in the Behavioural Inattention Test, conventional subtests (max. score 146, cut off 129)

| Patients | Lesion | Post-stroke (days) | Hemianopia | Neglect | BIT-Score (Cut off 129) |
|---|---|---|---|---|---|
| D.K. | R temporo-parietal | 1340 | No | Yes | 115 |
| E.R. | R fronto-temporal | 196 | No | Yes | 116 |
| L.C. | R occipital, inferior-temporal, basal ganglia | 570 | Yes | Yes | 115 |
| M.B.H. | R temporo-parietal | 857 | No | Yes | 125 |
| E.E. | R temporo-parietal | 197 | Yes | No | 133 |
| A.W. | R fronto-parietal | 633 | No | No | 135 |
| M.A.H. | R superior parietal | 195 | No | No | 140 |

### 2.4. *Dependent measures*

Kinematic and hand path parameters were calculated for each hand separately. Movement onset was defined as the first frame in which the wrist marker exceeded a velocity of 25 mm/s in the direction of movement. Movement end-point was defined as the first frame in which displacement of the target marker exceeded 1.0 mm in any direction. 1. Movement duration (MD) was defined as movement end-point minus movement onset. The following dependent measures were computed from the 3D co-ordinates for the markers placed on the thumb, index finger and wrist, and were used to analyse the kinematics of the grasp phase of the prehension task: 2. Peak grip aperture (PGA) between index finger and thumb (measured in mm). 3. The time taken to reach PGA as a percentage of total movement duration (TTPGA%). 4. To calculate a hand path curvature index (HPC-index) subjects' hand paths were spatially resampled and translated (see also Jackson et al. [15] for a description of this procedure). Spatial resampling was carried out to produce hand paths which each contained 100 equally spaced spatial segments, thereby allowing comparisons between movements. The spatial resampling did not change the shape of each individual hand path, and did not result in the normalisation of movement amplitude. Hand paths were also translated spatially so that movements to different target locations within the workspace could be compared. This procedure resulted in a set of hand paths aligned along a single axis. The HPC-index consisted of the ratio between the magnitude of the maximum lateral deviation achieved at any point during the movement (mm), and the straight line joining the kinematically-determined start and end positions of the movement (mm). Note that the HPC-index produces a measure of hand path curvature that is (a) independent of movement amplitude, and, (b) in which all values, regardless of whether the hand path curved leftwards or rightwards, are positive. As leftward and rightward movements can often show a roughly mirror symmetric curvature, we also carried out analyses to examine the sign of the hand path curvature (HPC) in both the controls and patients, assigning positive values for rightward curving hand paths and negative values for leftward curving hand paths.

The following dependent measures were computed from the 3D co-ordinates for the wrist marker, and were used to analyse the kinematics of the transport phase: 5. Peak velocity in the direction of movement (PV) and, 6. the deceleration phase measured as the time from peak velocity to endpoint as a percentage of the total movement duration (DT%).

### 2.5. *Statistical analyses*

Two sets of ANOVA's were performed on each of the dependent measures. The first set compared the RCVA group with the controls using their right hand (mixed 4-way ANOVA with Group, Loop, Hemispace and Distance as the factors). For the final analysis the RCVA group was further split into patients with acute neglect (RCVA N+) and the rest (RCVA N−) and again compared to the controls using their right hand. Since this ANOVA yielded no differences between patients with and without neglect on any of the dependent measures, these results will be reported in a complementary manner only.

## 3. Results

### 3.1. *Trajectory and grasp phase kinematics*

#### 3.1.1. *Peak Grip Aperture* (*PGA*)

Overall the maximum grip aperture of the patients (means: RCVA N+ $= 93$ mm, SD $= 10.8$; RCVA

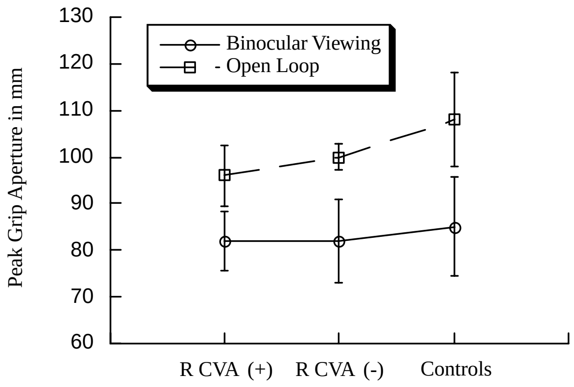

Fig. 1. Overall Peak Grip Aperture for the R CVA patients with and without neglect (R CVA (+), R CVA (−)) and the control group using their right hand. Error bars indicate SE's.

N− = 93mm, SD = 10.6) did not differ from the aperture shown by the controls (mean = 97.3 mm, SD = 17.8) on any of the ANOVA's (Fig. 1). Only viewing condition affected the opening of the hand with the maximum grip aperture proving larger in the open than the closed loop condition for all subjects (RCVA's vs Controls, $F(1,10) = 58.9, p < 0.001$).

#### *3.1.2. Percentage of Time taken to reach PGA (TTPGA%)*

Similarly to PGA there were no group effects with regards to the time taken to reach maximum peak grip aperture on any of the ANOVA's (means: RCVA N+ = 76%, SD = 20.3; RCVA N− = 75%, SD = 12.4; Controls right hand = 84%, SD = 6) and no other effects were found.

An additional attempt was made to ensure that the grasp kinematics were indeed unimpaired, by assessing the consistency and spatial accuracy of the subjects' grip orientation at the end of the reach. Since no group effects were found for the different viewing conditions in relation to the grip aperture parameters, this was done for binocular viewing only. The angle of the opposition axis formed between the index finger and the thumb was calculated one sample (20 ms) prior to contact with the target object. This opposition axis was calculated by taking the angle formed between the XY position of the thumb marker and the XY position of the marker located on the index finger.

In line with the PGA data no group effects were found (means: RCVA N+ = 48°, SD = 11; RCVA N− = 50°, SD = 13, Controls right hand = 40°, SD = 9) although there was a general effect of hemispace ($F(1,10) = 113.06$) with contralateral reaches giving rise to larger opposition angles (mean: 52°, SD = 12) than ipsilateral reaches (mean: 35°, SD = 10).

#### *3.1.3. Hand path curvature (HPC) and hand path curvature index (HPC-index)*

Looking at the hand path curvature (HPC) is was found that grasping movements made with the right hand resulted in rightward curvatures. This proved the case for patient and control groups over all locations and distances (Fig. 2).

With regard to the HPC-index, the ANOVA comparing the RCVA group with the controls revealed a significant Group by Viewing Condition interaction ($F(1,10) = 6.9, p = 0.02$). Further analyses showed that the RCVA group showed curvature of similar magnitude on both open and closed loop conditions whereas the controls showed significantly less curvature under binocular viewing than open loop conditions (Fig. 3). RCVA N+ and RCVA N- patients showed no differences with regard to the magnitude of curvature (RCVA N+ = 0.07, SD = 0.03; RCVA N− = 0.08, SD = 0.03).

There was also a main effect of hemispace ($F(1,10) = 54.75, p < 0.001$) with contralateral reaches revealing less curvature than ipsilateral reaches and a distance effect ($F(1,10) = 27.1, p < 0.01$) with near targets giving rise to larger curvature than far targets. These effects were shown equally by patients and controls.

### *3.2. Velocity analyses*

#### *3.2.1. Movement Duration (MD)*

Overall, the reaches of the RCVA patients took much longer than those performed by the controls (main effect of group, $F(1,10) = 21.6, p = 0.009$), see Fig. 4. There were, however, no significant differences in movement duration between the RCVA N+ whose mean duration time was 1057msec (SD = 186) and the RCVA N− group who showed a mean duration

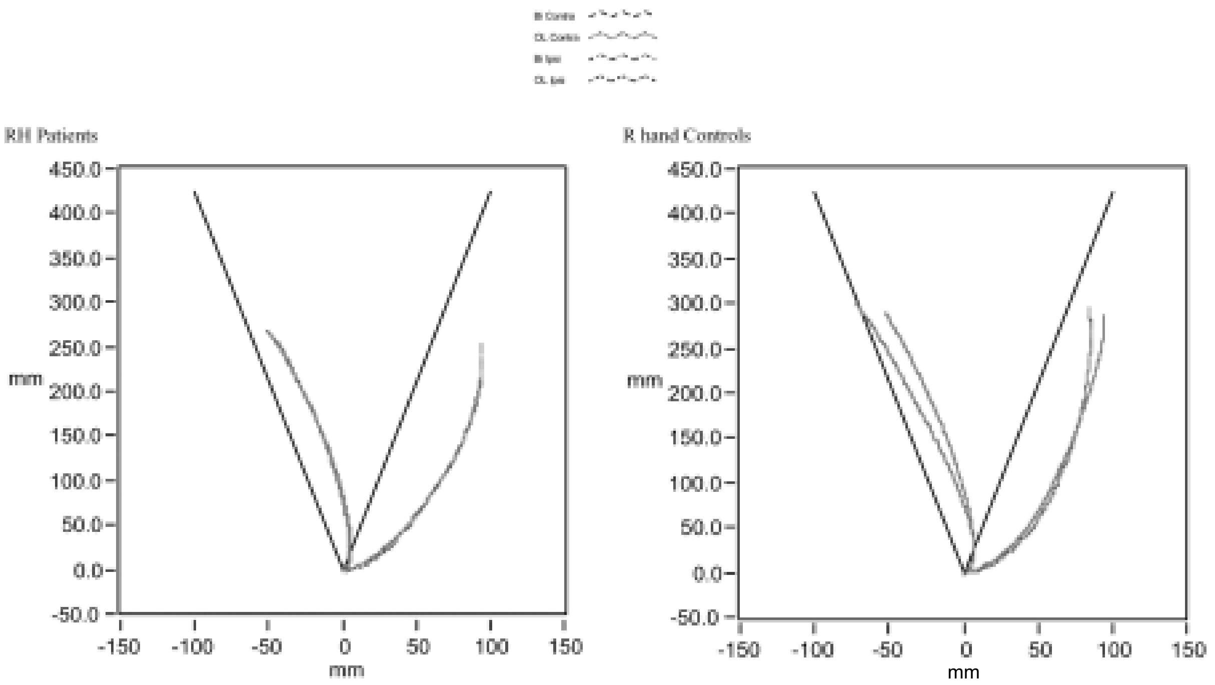

Fig. 2. Mean Hand paths for the control subjects and the R CVA patients (RH patients) plotted separately for viewing condition (Bi = Binocular, OL = Open Loop) and hemispace).

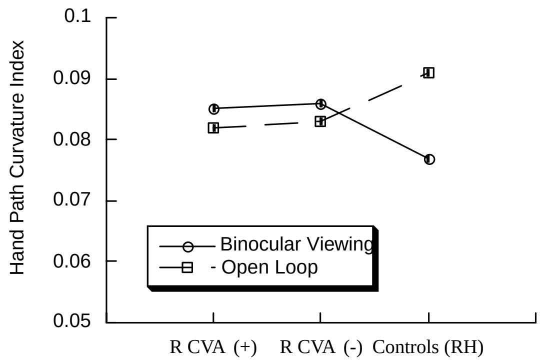

Fig. 3. Mean hand-path -curvature-index (HPC-index) for control subjects and RCVA patients (with and without neglect) under binocular and open loop viewing conditions. See text for HPC-index calculation. Error bars indicate SE's.

time of 1205msec (SD = 226), although both groups were significantly slower than the control group (mean 672 msec, SD = 168), Tukey-test.

There were also main effects of space (1,10) = 12.6, $p = 0.006$) and distance ($F(1, 10) = 85.7, p < 0.001$) with ipsilateral reaches taking less time than contralateral reaches and far reaches taking longer than near reaches.

### 3.2.2. *Peak Velocity* (*PV*)

Overall, the reaches of the controls reached much higher peak velocities than those performed by the RCVA patients (main effect of group, $F(1, 10) = 24.2, p = 0.008$). There were, however, no significant differences between the RCVA N+ whose mean peak velocity reached 480 mm/sec (SD = 89) and the RCVA N− group who showed a mean peak velocity of 478 mm/sec (SD = 89) although they were both significantly slower than the control group (mean 919 mm/sec, SD = 198), Tukey-test.

There was also a Group by Distance interaction ($F(1, 10) = 5.9, p = 0.03$). Further analyses revealed that target distance had no effect on the RCVA patients whereas the controls reached significantly higher peak

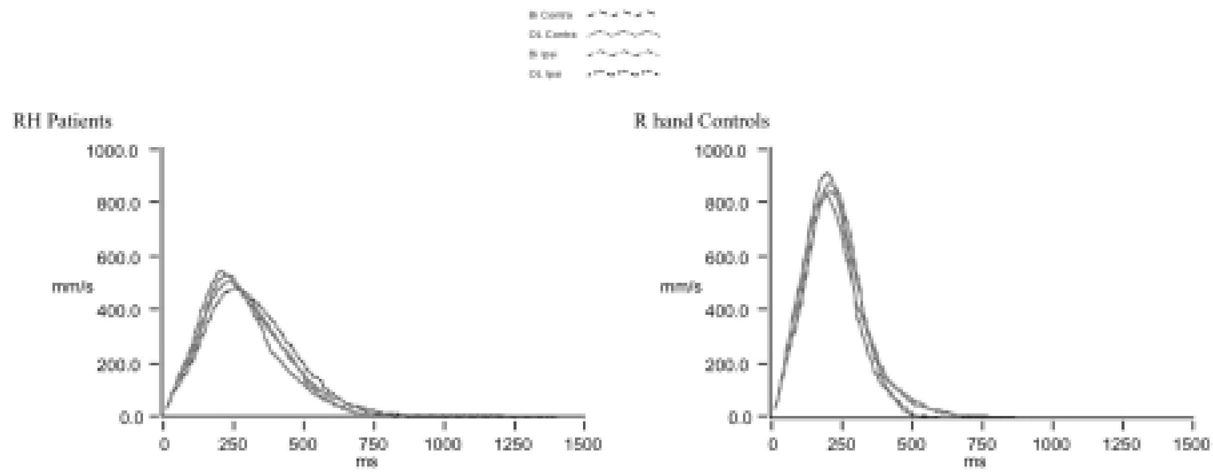

Fig. 4. Mean Velocity profiles for control subjects and RCVA patients (RH patients) plotted separately for viewing condition (Bi = Binocular, OL = Open Loop) and hemispace (Contra = Contralateral, Ipsi = Ipsilateral).

velocities for the far targets. There was also a Loop by Hemispace interaction ($F(1, 10) + 7.6, p = 0.02$). Planned contrasts showed that ipsilateral reaches led to higher velocities than contralateral reaches. Additionally contralateral reaches proved faster in the open loop than the binocular viewing condition. This was not the case for ipsilateral reaches.

### 3.2.3. *Percentage of Time spent Decelerating* (*DT%*)

The ANOVA comparing the RCVA group with the controls using their right hand revealed a significant Loop by Distance by Group interaction ($F(1, 10) = 11.76 p = 0.006$). Further analyses showed that the target distance and viewing condition had no effect on the time spent decelerating by the patients. Target distance did also not affect the controls. The controls, however, spent significantly less time decelerating under binocular as opposed to open loop viewing conditions (Fig. 5). There was also a main effect of hemispace ($F(1, 10) = 7.1, p = 0.02$) with less time spent on deceleration in contralateral space.

### 3.3. *Summary of the main results*

As the main motivation of this study was to investigate the effects of brain damage on the control of grasping, discussion of the results will mainly focus on the reported group effects:

It was shown clearly that neither of the two patient groups differed from the control groups in relation to the peak grip aperture or the grip orientation shown nor the time taken to reach the peak grip aperture. There was, however, an effect of path curvature in that right hemisphere lesioned patients showed the same amount of curvature with and without visual feedback, whereas the trajectory of the controls proved straighter in the closed loop condition. There was, however, no difference between patients with and without neglect with regard to the extent of curvature shown.

Both patient groups proved markedly slower on the velocity measures of MD and PV although there were no differences in the velocity profiles of RCVA patients with or without acute neglect. DT% revealed an interesting Group by Loop effect. Whereas the control group spent less time decelerating under binocular as opposed to open loop viewing conditions, this was not the case for the RCVA group suggesting that they might be less efficient in using visual guidance to home in on the target, an interpretation that also fits with the lack of curvature differences found between presence and absence of visual feedback in these patients.

## 4. Discussion

### 4.1. *Grasp kinematics*

The most interesting result of this study was the finding that neither peak grip aperture nor the time taken to reach peak aperture was impaired in any of the patient groups. Additionally grasp orientation was also normal. In line with Paulignan et al. [29] we found a significant hemispatial effect with contralateral reaches producing larger opposition axis angles than ipsilateral reaches for both subject groups. The authors suggest that prehension movements aimed at cylindrical objects

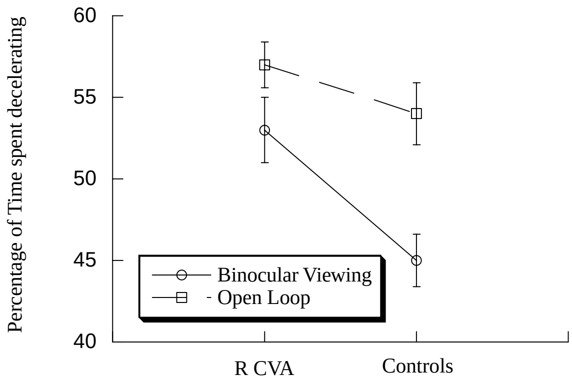

Fig. 5. Percentage of Time spent decelerating under binocular and open loop viewing conditions, separately for the R CVA patients (with and without neglect) and the control subjects using their right hand. Again, error bars indicate SE's.

are organised to minimise changes in the posture of the lower arm and that this position is thus not determined with respect to (external ) visual co-ordinates but mainly with respect to body centred co-ordinates.

These three findings clearly show that neglect patients have no problems in scaling their grip size when reaching to objects placed in near and far locations, both in left and right hemispace. Although similar results have already been reported [4,32], we now report this in a group of neglect patients and also extend the finding to grasp orientation and grasping movements executed in the absence of visual feedback. The possibility that visuomotor grip scaling might be normal under open and closed loop conditions in neglect patients clearly dissociates this syndrome from the observed cases of optic ataxia [16,31] and also directly contrasts with the behaviour of patient AT reported by Jeannerod et al. [19]. This patient presented a bilateral deficit in grasping simple objects, both in the presence and absence of visual feedback, without a concurrent deficit in reaching towards the location of these objects.

It is tempting to link the observed dissociations to varying lesion sites within the parietal lobe. Both Milner and Goodale [27] and Perenin [30] have recently argued that lesions resulting in optic ataxia tend to lie in the upper part of the parietal lobe around the intraparietal sulcus but that the focus for neglect lies more ventrally, in the region of the supramarginal and angular gyri. Indeed the previously described study by Binkofski et al. supports this: only patients whose lesions included the intraparietal sulcus showed abnormalities in grasping, whereas in the three patients in which this area was spared no abnormalities were found. However, although this distinction might be generally acceptable, no conclusive interpretations can be made when other grasping studies are analysed. Although the optic ataxia patients tested by Perenin and Vighetto [31] had lesions mainly confined to the superior parietal lobe, both Jeannerod et al.'s [19] and Jakobson et al.'s [16] patients had lesions in both the superior and inferior parietal lobes and although the lesions were not mapped, we can assume that this was also the case for some of the patients presented here. Chieffi et al.'s [4] patient had a subcortical and Pritchard et al.'s [32] patient an occipito-temporal lesion. It is thus hard to make specific anatomical predictions from such diverse data and probably more useful to look at the findings in terms of function:

Although hemispatial neglect is typically described as a disturbance of spatial behaviour, the main problem is a failure to attend to or represent objects presented in the contralesional space. This then results in a variety of symptoms, i.e., ignoring part of the objects completely [5,39], misrepresenting their size [2,28] or failing to explore or localise them adequately in space [10,21]. However, these problems may not be directly related to goal-directed behaviour. The programming and on-line control of a particular action typically requires a unique set of transformations of the visual array, so that each component of the action can be correctly executed with respect to the goal object. Thus, to fixate and then reach towards a goal object, it is necessary that the location and motion of that object be specified in egocentric co-ordinates (that is, coded with respect to the observer). To form the hand and fingers appropriately for the grasp, the coding of the goal object's shape and size would also need to be largely viewer based. Finally, since the relative position of the observer and the goal object will change, it is obvious that the egocentric co-ordinates of the object's location and its surface and contours must be computed on each occasion. It is possible that neglect may not operate within these broad co-ordinates of egocentric space, but within the internal co-ordinates of objects or figures themselves. One interesting example of this internal object representation is the metric relating sizes to weights. This ratio determines expectations when lifting objects and there is recent evidence from normal subjects that grip force miscalibrations can occur when observers are deceived about the size of objects under the influence of the 'Ponzo' illusion [14]. However, the experiment also showed that the opening movements of the fingers prior to contact with the object were not miscalibrated. This same dissociation holds true for some neglect patients. Shaw et al. [36] who studied grip force calibrations in such patients found that, compared to controls, neglect patients dramatically overgrip objects, especially those presented in left space. Two of the patients tested in that study (LC and DK) were also included in this experiment and showed no miscalibration of their hand opening in either right or left space. Although these data seem to suggest that neglect patients might be less impaired in egocentric as opposed to allocentric coding, the path curvature data of this experiment do not entirely fit with that interpretation (see below).

A caveat also has to be made regarding the fact that in this experiment object size was not explicitly manipulated. The subjects therefore did not have to rely on the visual size of the object for the scaling of their hand as indeed they could have used kinaesthetic memory of the object size. It thus has to be granted that the lack of abnormalities of the grasp components may have been a result of the experimental design. However, our data are in accordance with two previous studies [5,34] who did manipulate object size but also found no abnormality in the grasp aperture.

### 4.2. *Path curvature*

The main finding with respect to reach trajectory was the group by loop interaction: right hemisphere lesioned patients showed the same amount of curvature with and without visual feedback, whereas the trajectory of the controls proved straighter in the closed loop condition. Reach trajectories did, however, not differ between patients with and without neglect. The findings of the controls are in line with Sergio and Scott's [35] data who also found significantly decreased path curvature when subjects grasped with visual feedback as opposed to a blindfolded condition. They give two possible explanations for this effect, one stating that hand trajectory may be explicitly defined in the series of sensorimotor transformations involved in converting visual target information into motor output to muscles, although, as they also argue, the neural correlate of this seems elusive. Their other explanation maintains that hand trajectory is determined largely at a perceptual rather than motor level. This argument is supported by the findings that with the availability of visual feedback of the hand, subjects maintain relatively straight hand trajectories at the expense of curved joint trajectories and vice versa when vision is available for the joint trajectories [7]. This explanation seems also likely to hold for the data presented here. It is possible that the right hemisphere lesioned patients were less efficient in using the visual feedback possibly due to their distorted topography of the visual representation. The lack of visual feedback may have thus affected them less than it did the controls. This relative increase in curvature in comparison to the control subjects is also consistent with the Jackson et al. [15] finding who reported that three patients recovering from neglect showed significantly more curved trajectories to visually over proprioceptively defined targets.

On the surface, our data also seem consistent with Chieffi et al.'s [4] result of a normal trajectory towards single targets in conditions with visual feedback. Simply looking at the closed loop condition we also found this, as there was no group effect. The interaction effect however, is at odds with other studies. Goodale et al. [9] found increased rightward curvature in closed loop pointing. We also found this but not when comparing the trajectory to control data but by comparison to the open loop condition. Both Harvey et al. [12] and Karnath et al. [22] found that controls show similar reach trajectories in conditions with or without visual feedback. Karnath et al. found this pattern in the patient subjects as well, whereas Harvey et al. found right hemisphere lesioned patients to make large rightward deviations under open loop. We found that our control subjects made straighter reaches when vision was available, whereas the trajectories of the right hemisphere lesioned patients were curved to the same degree in both conditions. Part of the differences in these studies might be explained by physiological findings that reaches and grasps may rely on different neural subsystems [24,37] and it may thus not be appropriate to compare pointing and grasping experiments directly. This does, however, not explain the discrepancies within the pointing experiments. These discrepancies might boil down to differences in the individual experimental setup. Maybe illumination was much lower in the Harvey et al. [12] than the Karnath et al. [22] open loop condition, which might have resulted in a larger disorientation in their patients. It is possible that the pointing condition in the Goodale et al. [9] study was somehow harder than the one used in the other two studies. One of the things that can be said, though, is that even if a bias in the trajectory occurs, there is no evidence so far that this bias is typical for neglect patients only. Like Karnath et al. [22] we found no differences in the trajectories of our neglect patients when compared to the right hemisphere lesioned controls and Harvey et al. [12] found a bias in right hemisphere lesioned patients without neglect. It thus seems that if there is an impairment in goal-directed behaviour it is not confined to hemispatial neglect.

However, unlike the two studies mentioned here [12, 22] our experiment had a relatively small number of patients which might have contributed to the lack of differential effects. Further, although there were clear differences between the two patients groups in terms of their neglect behaviour (the four neglect patients only ever omitted items on the left whereas the three control patients omitted items more generally in both left and right space and less frequently overall), it has to be granted that the neglect patients did not exhibit very severe neglect scores and the control patients were not perfect on this test. These could well be contributing factors to the fact that no differences were found between these two patient groups and in future it may well be informative to test patients with more severe neglect.

So bearing these caveats in mind, when comparing these right hemisphere lesioned patients to controls what we seemed to have found are unimpaired grasp as well orientation parameters, whereas the path curvature seems to be relatively greater under visual feedback. This latest parameter thus seems the most sensitive in reflecting any perceptual misrepresentations these patients may experience possibly because (as argued by Jackson et al., [15]) at least initially, the trajectory is less driven by a monitoring of the moment by moment changes on the hand relative to the target. Thus movements may be anchored at both the beginning and the end of the movement but less so during mid reach when they may be maximally susceptible to misrepresentations.

### 4.3. *Velocity profiles*

Both patient groups proved markedly slower on the velocity measures of movement duration and peak velocity although there were no differences in the velocity profiles of RCVA patients with or without acute neglect. Not many studies looking at goal-directed behaviour in brain lesioned patients have reported velocity profiles as well as reach trajectory and grasp kinematics. No mention of temporal kinematics is made in the Goodale et al. [9], Karnath et al. [22] or Pritchard et al. [32] studies. Chieffi et al.'s [4] patient showed normal execution time but then it was only a single case. Harvey et al. [12] again, did not report movement time. The temporal kinematics were, however, analysed in these patients [11] and comparable to the presented data, movement time and peak velocity proved longer and lower for both RCVA and LCVA groups when compared to the control subjects. This was also found in two other studies by Konczak and Karnath [23] and Hermsdörfer et al. [13] whose patients were slowed in all movement kinematics although again there were no differences between patients with and without neglect. The main finding is that these slowings are not directionally specific, i.e., there is no relative slowing in movements towards leftwardly compared to rightwardly located targets (this was not explicitly analysed by Hermsdörfer et al. [13]). Also in the present study, there was no contralesional slowing beyond that shown by the control group in terms of movement duration and peak velocity, a general effect that is certainly due to mechanical constraints (see [3]). It thus seems that patients with right damage exhibit a general slowing of their kinematics but that this bradykinesia does not seem to be directionally specific towards targets located in contralesional space.

Additionally to the general slowing of arm movements in both patient groups, RCVA patients with and without neglect proved specifically impaired with respect to deceleration time: the control group spent less time decelerating under binocular as opposed to open loop viewing conditions, suggesting on-line use of visual feedback. This was not the case for the RCVA group indicating that they may be less efficient in using visual guidance to home in on the target, an interpretation that also fits with the lack of curvature differences found between presence and absence of visual feedback in these patients. Hermsdörfer et al. [13] also reported prolonged deceleration times in their right brain damaged patients compared to the control groups and there is a trend for prolonged deceleration in Konzcak and Karnath's [23] data a well.

However, these data are different from those of Fisk and Goodale [6,8] who demonstrated no impairment on any of the kinematic measures for their right hemisphere damaged subjects, while pointing under closed loop conditions. Nonetheless, the results presented here indicate a right parietal involvement during the adjustment phase, suggesting a less efficient use of the available visual feedback. If one accepts the assumption that the RCVA patients had difficulty in determining the target position (see also path curvature, above), it is possible that the initial direction of the movement while accelerating was less driven by a monitoring of the moment by moment changes of the hand relative to the target and consequently modifications of the trajectory would need to occur during deceleration. However, rather than making efficient use of the visual information provided, RCVA patients probably reached the object by falling back on the initial representation they had of that object in space. This would explain the lack of differences in both deceleration time and path curvature between binocular and open loop viewing conditions compared to the binocular advantages shown by the healthy controls.

As with all the other parameters no differences were found in the velocity profiles of patients with and without neglect. This is surprising in the light of a whole range of studies that have found neglect patients to be specifically impaired while reaching into left space or reaching towards positions relatively leftwards of the position the hand is initially placed at (see [25] for review). However, all these studies may not be directly comparable to prehension studies as there is always more than one stimulus presented. This may lead to competition of input as well as output parameters in these patients and may require more complex attentional and representational mechanisms than those elicited by a simple goal-directed reaching or grasping response towards a single target.

## Acknowledgements

We would like to thank all the patients and other participants for their co-operation and the two anonymous reviewers for very insight and helpful comments on a previous version of this manuscript. This work was supported by Wellcome grants to M Harvey (No: 050184/Z) and SR Jackson (No: 054665). T Krämer was supported by a DAAD scholarship.
